# Past and Present of Electrochemical Sensors and Methods for Amphenicol Antibiotic Analysis

**DOI:** 10.3390/mi13050677

**Published:** 2022-04-27

**Authors:** Iulia Gabriela David, Mihaela Buleandra, Dana Elena Popa, Mihaela Carmen Cheregi, Emilia Elena Iorgulescu

**Affiliations:** Department of Analytical Chemistry, Faculty of Chemistry, University of Bucharest, Panduri Av. 90-92, District 5, 050663 Bucharest, Romania; mihaela.buleandra@g.unibuc.ro (M.B.); elena.popa@chimie.unibuc.ro (D.E.P.); emilia-elena.iorgulescu@chimie.unibuc.ro (E.E.I.)

**Keywords:** amphenicol, chloramphenicol, thiamphenicol, florfenicol, sensor, electrochemical detection, modified electrode

## Abstract

Amphenicols are broad-spectrum antibiotics. Despite their benefits, they also present toxic effects and therefore their presence in animal-derived food was regulated. Various analytical methods have been reported for their trace analysis in food and environmental samples, as well as in the quality control of pharmaceuticals. Among these methods, the electrochemical ones are simpler, more rapid and cost-effective. The working electrode is the core of any electroanalytical method because the selectivity and sensitivity of the determination depend on its surface activity. Therefore, this review offers a comprehensive overview of the electrochemical sensors and methods along with their performance characteristics for chloramphenicol, thiamphenicol and florfenicol detection, with a focus on those reported in the last five years. Electrode modification procedures and analytical applications of the recently described devices for amphenicol electroanalysis in various matrices (pharmaceuticals, environmental, foods), together with the sample preparation methods were discussed. Therefore, the information and the concepts contained in this review can be a starting point for future new findings in the field of amphenicol electrochemical detection.

## 1. Introduction

Antibiotics are among the most used pharmaceutical compounds for human and veterinary medicine, with multiple benefits. They have mainly therapeutic and prophylactic actions, or they are administered to food producing animals in order to stimulate their growth and to increase productivity [1]. Nevertheless, the abuse of antibiotics and implicitly their presence in animal origin food have side-effects on human health. Thus, some pathogens become resistant to antibiotics, allergic reactions can appear and dysfunctions of various systems in the human body can occur [2]. Moreover, antibiotics and their residues pose an ecological risk due to their toxicity as they can be found in aquatic environments [3].

**Figure 1:**
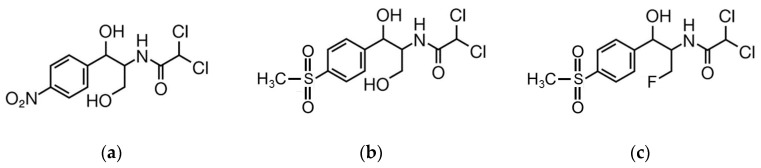
Chemical structure of (**a**) chloramphenicol (CAP); (**b**) thiamphenicol (TAP); (**c**) florfenicol (FF).

Amphenicols are broad spectrum synthetic antibiotics, with activity against Gram-positive and Gram-negative bacteria, also acting efficiently towards anaerobic microorganisms and viruses [4]. The action mechanism of phenicols is based on their adsorption on bacterial cells and inhibition of protein synthesis by binding to ribosomal subunits [4]. This antibiotic family includes small lipid-soluble organic molecules, namely, chloramphenicol (CAP), thiamphenicol (TAP) and florfenicol (FF) (Figure 1).

CAP was initially isolated from *Streptomyces venezuelae* and named chloromycetin, but now is often chemically synthetized. It has excellent pharmacokinetic and antibacterial properties, proved in the past by extensively using CAP in veterinary medicine, but nowadays, it is found as ophthalmic solutions applied to treat some human eye infections (conjunctivitis) and as tablets used against meningitis, typhoid fever and different respiratory and nervous systems infections [2]. 

Despite all recognized benefits and of the fact that the production of this antibiotic is cheap, the presence of the nitrobenzene moiety in CAP structure makes it toxic for humans [5]. The adverse effects include headache, nausea, numbness in hands, bone marrow aplasia (loss of ability to produce blood cells) and therefore aplastic anemia, cardiovascular collapse, inhibition of the cytochrome P450 2C9 and 3A4 isoenzyme and gray infant syndrome can arise [5,6]. Moreover, when CAP is administrated for a long time, even in very small doses, it may lead to *Escherichia coli* and *Salmonella* drug-resistance [7]. For this reason, CAP has been banned in many countries for use in the treatment of food-producing animals. For example, the EU has forbidden the product since 1990 by including it in Annex IV of Regulation No. 2377/90 [8], currently being listed as a prohibited substance in Commission Regulation No. 37/2010, for which MRLs cannot be established [9]. 

An issue that can be underlined refers to the obviousness that CAP is a well-studied protein synthesis inhibitor, representing the basis for obtaining derived compounds with similar benefits and minimal side effects [10]. Thus, TAP and FF are structural analogs of CAP; TAP is a second generation CAP derivative with a methane-sulfonyl group instead of para-nitro group and FF is a fluorinated derivative of TAP (third generation), in which the hydroxyl group at C3 is replaced with fluorine [11]. Due to the fact that TAP and FF are less toxic than CAP, but exhibit analogue antibacterial mechanisms, they successfully replaced CAP for veterinary use. It is worth mentioning that FF, in contrast to TAP, converts into some metabolites, the main one being florfenicol-amine that is considered a marker for FF use [12]. However, due to animal and human health concerns, MRLs have been established for both derivative compounds. Thus, in Regulation 37/2010 it is stipulated that the MRLs of TAP in foodstuffs of animal origin (muscle, liver, kidney, milk and fat) are 50.00 μg/kg. The MRLs for FF (calculated as the sum of FF and its metabolites measured as florfenicol-amine) in muscle, liver, kidney, skin and fat are between 100.00 and 3000.00 μg/kg (depending on the food matrix) [9]. Moreover, as a supplement to the provisions of European Regulation 470/2009 [13], FF and TAP are not recommended for animals from which milk or eggs are produced for human consumption [9]. 

In order to rigorously control the trace concentration of amphenicols in animal origin foods, sensitive and versatile analytical methods are needed. Moreover, for the analysis of prohibited substances, such as CAP, in Decision 2002/657/EC the European legislation provides the MRPL of the methods, also establishing performance criteria and procedure for the validation of analytical methods. Thus, the MRLP of CAP is 0.30 μg/kg and constitutes the minimum content which has to be detected and confirmed [14]. This justifies the necessity to develop methods that combine superior performance characteristics. 

Various analytical methods have been reported for the trace detection of amphenicols in complex matrices as foods, either as single or multiple analytes. These are based on separation techniques (gas and liquid chromatography, capillary electrophoresis) coupled with sensitive detectors (diode array, UV-Vis, fluorescence, MS) [15,16,17,18,19] and are used as confirmatory methods for analyte identification and quantification. Furthermore, the microbiological methods are considered suitable for screening purposes, these being reviewed for the detection of amphenicol residues in different types of foodstuffs (milk, meat, eggs, honey, seafood) [20]. Although these methods have undeniable advantages, they are expensive, time consuming, require additional sample preparation steps and are not applicable for on-site analysis. 

On the other hand, analytical methods based on electrochemical techniques are cost affordable due to reduced reagents consumption and less expensive instrumentation, simple and rapid, possess satisfactory performance characteristics and are suitable for miniaturization and portability, features that allowed them to be extensively applied for various matrices analysis in areas such as biomedicine, pharmaceutics, environment and food [21,22,23]. Among the advantages can also be listed: the possibility to be used in both colored and turbid solutions, the need for minimum sample processing and the ability to be applied in multi-element analysis. 

In order to obtain information regarding the electrochemical behavior of the analyte, the kinetics and thermodynamics of the electrode process, CV is the first choice and the main exploited tool. In addition, CV is commonly used for electrode surface modification by electro-polymerization or electrochemical activation and also to investigate the electrochemistry of modified sensors. To obtain complementary data about the modified electrode surface, a complex technique is used, namely, electrochemical impedance spectroscopy (EIS) that enables the study of the interfacial properties, exploring the processes implying mass or charge transfer and diffusion [24]. 

Regarding the quantitative determination of amphenicols, more sensitive voltammetric (DPV, SWV and AdSV) or amperometric techniques combined with the electrode performances allow attaining low quantification limits, meeting the criteria imposed for MRPLs and even wide linear ranges. The target analyte concentration can be determined as well as by measuring the voltage variation between the ion-selective electrode and the reference one by means of potentiometric techniques. Potentiometric sensors offer short response time, low cost and long lifetime as strengths. However, the “heart” of the electrochemical methods is the working electrode, which transduces the electrochemical response of the analyte into a signal, which is further transformed into analytical information by the electronic part of the measurement assembly. Thus, the working electrode practically establishes the performance characteristics of the methods. In order to perform analysis of various species found at low concentrations in complex matrices there is a continuous need to improve the sensitivity and selectivity of the sensors and this can be performed by modifying their surfaces with a variety of species. 

In the literature there are some recent reviews that provided overviews on the electrochemical detection of antibiotics [25], most of them being focused on certain topics, such as the sensing material [26,27,28,29] or the analyzed sample, e.g., milk [30]. However, strictly amphenicol electroanalysis was previously summarized in few papers, one published in 2013 about the electrochemical and immuno-sensing of CAP [31], one from 2016, which is related to amphenicol (electro)sensing [32] and the most recent ones, from 2018 that refer to aptasensors [33,34]. However, all these reviews emphasize the importance of developing new and more sensitive devices and methods for antibiotics monitoring in complex matrices. The present paper offers a comprehensive overview on the electrochemical sensors and methods for amphenicol detection, as it mentions all those developed over time, along with their performance characteristics, but it also contains completely new, more detailed, updates on those reported in the last five years (2017–2022). Some general issues regarding the electrode modification procedures, the detection mechanism and the analytical applications of the recently described devices for amphenicol electroanalysis are discussed, examples being given in each case. It is important to point out that we also briefly present aspects regarding the real sample (food, environmental, etc.) preparation steps preceding the actual electrochemical detection. This aspect is not commonly discussed in the literature despite the fact that the sample pretreatment procedure represents a key element in all analytical methods. Therefore, the information and the concepts contained in this review can be a starting point for future new findings in the field of amphenicol electrochemical detection.

## 2. Procedures for the Preparation of Electrochemical Devices for Amphenicol Antibiotic Analysis

**Figure 2:**
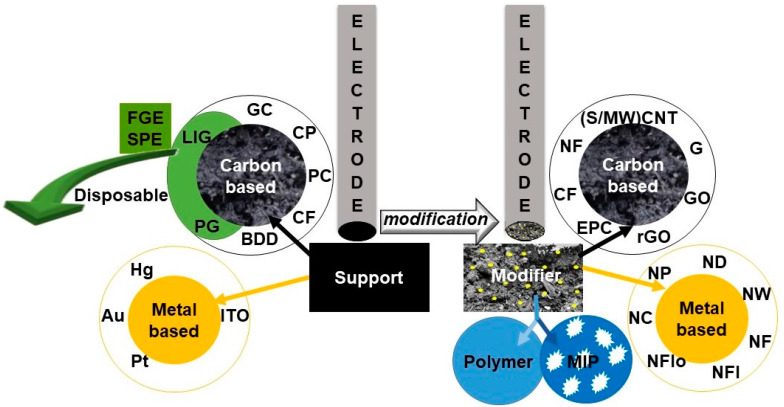
Illustration of electrode supports and modifiers employed in the sensors prepared for amphenicol analysis.

The number of developed sensors is increasing rapidly due to multiple modification procedures and the large amount of materials [25,26,27,28,29], employed either alone or in combination, in order to change the surface of various electrochemical supports. The specific characteristics of the species immobilized on the electrode surface resulted in improved sensitivity [27] and selectivity of the measurements. Figure 2 schematically indicates the electrode materials (support and modifiers) used for amphenicol electroanalysis and Table 1 summarizes the corresponding sensors along with the performance characteristics of the determination methods. 

One class of modifiers is represented by polymeric films which can be either chemically synthetized and subsequently used to cover the electrode or they can be directly electrochemically deposited at the electroactive surface. For example, sulfonated polyaniline was chemically synthetized starting from aniline and aminobenzenesulfonic acid, while sulfonated polyaniline and chitosan were co-electrodeposited at the GCE by applying a constant cathodic current [95]. Using CV, poly(Eriochrome black) [92], poly(methylthiophene) [119] and tetraruthenated porphyrin polymeric films [120] were deposited at the GCE. 

Sometimes the procedure used to change the surface characteristics involved more steps, such as in the case of the MIP(EBT)/SPCE preparation that followed the stages: (1) SPCE electrochemical cleaning by chronoamperometry (1.700 V, 180 s, 0.10 mol/L KCl); (2) SPCE covering with the electrogenerated conducting polymer PEDOT (0.900 V, 10 s, monomer: 3,4-ethylendioxythiophene) because it reduced the background signal, decreased the charge transfer resistance (R_ct_) and increased the peak current, generating highly stable currents; (3) incubation in 4-aminothiophenol in order to enable covalent links between the PEDOT and the subsequent Eriochrome black T polymeric layer, 4-aminothiophenol acting as a “cross-linker” due to its –SH group; (4) the chronoamperometric MIP(EBT) deposition (0.950 V; 250 s; template: CAP, monomer: Eriochrome black T in acetonitrile); (5) electrochemical removal of the template by multiple consecutive CV scans (−0.200 to −0.800 V) in acetonitrile using lithium perchlorate as supporting electrolyte [108]. 

CV was used not only to cover the electrodes’ surfaces with polymers but also to modify GCE with the hybrid material Z-800/rGO [56]. On the contrary, Fe_3_O_4_ was electrodeposited onto the GCEs by applying a fixed potential (–1.300 V; 180 s) to the electrode in FeCl_3_ solution [46] and gold nanoparticles were electrodeposited on the BDDE surface from a 0.10 mol/L H_2_SO_4_ solution containing HAuCl_4_ using a three (nucleation-growing) pulses procedure. A nucleation pulse consisted of 2 s at −0.700 V, followed by growth pulses of 0.000 V with the duration of 150 s, 300 s and 800 s, respectively [48]. 

Other electrochemical sensors developed for CAP analysis were obtained by drop casting [61,65,69,84,105,112,113] or drop coating methods. Thus, for example, in the first approach a thin film was formed by dropping an ethanolic graphene nanoflakes dispersion [50], a polydopamine functionalized vapor-grown carbon fiber composite slurry [94], DMF suspension of Co_3_O_4_@rGO [58] or of graphene/copper phtalocyanine [62], a solution containing gold nanoflowers, N-hydroxysuccinimide and graphene oxide [107], a dispersion in DMF of the nanocomposite carbon nanotubes/ethylene diamine/gold nanoparticles [118], Gd_2_(MoO_4_)_3_@rG slurry in isopropyl alcohol and Milli-Q water [57], ZnWO_4_ nanowires [90] or GO/PdNPs [66] ethanolic suspension, dispersion of Eu_2_O_3_NPs@rGO in water:ethanol [111] or of Fe/NC in deionized water:isopropyl alcohol:Nafion [88] on the electrode surface. This step was followed by drying in ambient conditions [50,57,65,66,88,94,105,107,111,113], at higher temperatures [59,61,84,112] or under vacuum [62]. 

Drop-coating method was employed to prepare Mn_2_O_3_TNSs/SPCE [110], MIO@NG/MSPE [115] and Ag/CMC@TiO_2_/LIGE [117] or to modify GCE with rGO/PdNPs [67], N-PC@MoS_2_ [82], MIL-101(Cr)/XC-72 [85], Cl-rGO [55] and g-C_3_N_4_/MnWO_4_ [89]. 

In some situations, the change of the electrode surface with various materials involved successive modification steps combining different methods. For example, a GCE was modified with multiwalled carbon nanotubes by drop-coating and subsequent electrodeposition at constant potential (−0.500 V; 6 s) of copper nanodendrites from CuSO_4_ solution [72]. Similarly, ordered mesoporous carbon@polydopamine suspension was dropped on the GCE and the modified electrode was peroxidized by CV (NaOH, −1.250 to 0.800 V, 0.050 V/s) in order to increase the negative charge on the polydopamine surface so that the electrostatic attraction between the polymer and CAP molecules was also enhanced through the positively charged analyte in the solution. On this electrode β-cyclodextrin was subsequently electropolymerized by CV (0.10 mol/L phosphate buffer solution pH 7.00, 8 cycles, −2.000 to 2.500 V, 0.100 V/s) [93]. Poly(3,4-ethylendioxythiophene) was synthesized by CV (−0.200 V to 1.200 V; 0.500 V/s) on indium tin oxide electrode in acetonitrile containing the monomer 3,4-ethylendioxythiophene and the supporting electrolyte C_8_H_20_NClO_4_. The PCN-222-CHIT/PEDOT/ITOE was prepared by dropping the mixed solution of PCN-222 and chitosan on PEDOT/ITOE surface and drying [97]. The preparation of MIP(MAA)/3D_CNTs@CuNPs/GCE included several steps: (1) carbon nanotubes functionalization with amino groups to generate reaction centers for the deposition of copper nanoparticles to obtain the 3D_CNTs@CuNPs composite; (2) drop-coating of this composite water dispersion onto the GCE surface and drying at room temperature; (3) chemical synthesis of the molecularly imprinted polymer using methacrylic acid as monomer and CAP as template molecule followed by (4) its drop coating at the previously obtained 3D_CNTs@CuNPs/GCE and drying at room temperature [74]. In order to prepare the MIP(5-IAA)/(Pt-Pd)NPs/P N-G/GCE, an aqueous suspension of Pt-Pd bimetallic nanoparticles and porous N-doped graphene was dropped onto GCE and after drying, the electrode was electrochemically covered with a molecularly imprinted polymer obtained by CV performed in a solution containing FF as template molecule, indole-5-carboxylic acid as monomer and tetrabutylammonium perchlorate as supporting electrolyte. The template molecule was removed from the polymer by keeping the electrode in a methanol:acetic acid solution [121]. 

A more complex procedure was described for the preparation of Apt-MIP(Res)/AgNPs/3-ampy-rGO/GCE that involved first covering the GCE with reduced graphene oxide functionalized with 3-aminomethyl pyridine followed by the attachment of silver nanoparticles through formation of Ag-N bounds with the -NH_2_ groups of 3- aminomethyl pyridine. The thus modified GCE was drop coated with the aminoaptamer [CAP] complex and thereafter covered with the molecularly imprinted polymer electrochemically synthetized by CV using resorcinol as monomer. CAP molecules were removed from the electrode surface by keeping the sensor in the washing solution (phosphate buffer solution:acetic acid:ethanol:acetonitrile) in the presence of sodium dodecylsulfate [99]. 

It is worth mentioning that some reports indicated that the GCE was electrochemically pretreated before the modifier deposition, e.g., by CV (−0.400 to 1.500 V; 0.100 V/s; 25 cycles; in 0.10 mol/L H_2_SO_4_), afterwards being subjected to a second identical pretreatment in 0.10 mol/L KOH [92] or by applying a fixed potential of 0.900 V for 60 s followed by CV (–1.000 to 0.600 V, 0.050 V/s; 10 cycles; 0.10 mol/L KCl) [46]. In the case of the rGO@NHS@AuNFlos/SPE the CV (–1.400 to 0.700 V, 0.100 V/s, 10 cycles) was performed after SPE modification [107]. 

The development of wearable sensors or sensor platforms has gained even more interest in recent years. Li and Bo [116] developed a three flexible graphene electrode system as a wearable electrochemical sensor which can be fixed on a finger and enables the rapid real-time on site measurement of CAP, clenbuterol and ractopamine in meat or milk by touching the contaminated sample. The system consisting of three flexible graphene electrodes, one being the working electrode, one the counter electrode and one being electroplated with Ag to act as reference electrode, was patterned and prepared at ambient conditions by irradiation of a commercially available polyimide film with a computer-controlled CO_2_ laser source, which produced the multilayer graphene with a 3D network through a photothermal reaction. The thus obtained graphene layer can be removed from the polyimide film by ultrasonication. The three flexible graphene electrode system was glued together with a finger of a disposable nitrile glove and connected to a portable electrochemical analyzer that transmitted the measured signal to a smartphone via Bluetooth. This flexible graphene electrode system can detect the analytes either in the solution or in solid forms. If the solid is a powder, in order to bring it in a measurable form, this must be transformed into a liquid by hydrogel casting on the electrode system surface. 

Another small (1.00 × 2.50 cm^2^) three electrode system for CAP analysis [122] was also produced by using a computer-controlled CO_2_ laser machine to engrave the graphene working and the counter electrodes on a polyimide substrate. The reference electrode and the conductivity tracks were obtained by a thermal process using silver conductive ink. The parts of the device which must not be in contact with the analyzed solution were isolated by encapsulation with plastic sheets. To obtain the molecularly imprinted poly(Eriochrome black T) laser induced graphene electrode, the working electrode was modified with molecularly imprinted poly(Eriochrome black T) according to the previously described procedure [108]. 

In order to attain the best electroanalytical performances of a chemically modified electrode, besides following various modification stages necessary for its preparation, the development of such sensors also involves the optimization of the conditions for each individual step. This is usually carried out by comparing, after each step, the analyte signal obtained at the modified electrode with that recorded in the same conditions at the bare one. It was experimentally demonstrated that the amphenicol electrochemical response increases with the thickness of the modifier(s) layer deposited on the electrode surface due to the increased conductivity and surface area, but an excess of covering material resulted in a decrease of the analyte signal because thicker layers hinder the electron transfer to the electrode surface [51,55,67,89,92]. If a polymer or a molecularly imprinted polymer is electrodeposited at the electrode surface then its thickness depends on the monomer (and the template, in the case of molecularly imprinting) concentration and on the electrochemical deposition conditions, e.g., number of cycles in the case of CV [92,93,119] or applied potential and time when chronoamperometry is applied [108], and these parameters must be optimized. If the polymeric material is chemically synthetized, then its composition and the amount (that depends on the concentration and the volume of the suspension) loaded on the electrode surface must be optimized [74,103]. Similarly, when the electrodes were covered with various other simple or hybrid materials the composition of the composite [66] as well as the quantity of the modifier at the sensor surface were studied in order to establish the best conditions for the amphenicol quantification [51,55,61,67,72,89,93,111,112,117,118].

## 3. Principles of Amphenicol Electrochemical Detection

### 3.1. Electrochemical Reduction

**Figure 3:**
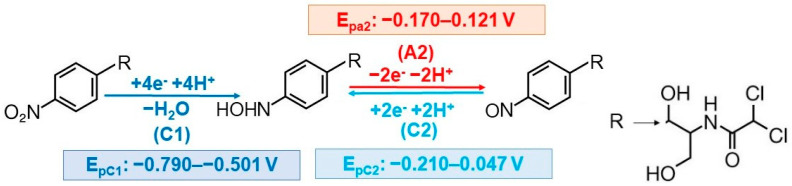
Schematic representation of CAP general electroreduction mechanism.

There are at least two main factors that influence the electrode reactions of an analyte, namely, the (i) type of the electrode electroactive surface and (ii) the pH and composition of the supporting electrolyte. CAP electrochemical behavior was investigated at various metallic or carbon-based electrodes, which were used as such or their surface was modified with different materials (Figure 2) in order to improve the sensitivity and selectivity of the determination. In most cases CAP (volt)amperometric detection was based on the reduction of the –NO_2_ group existing in its structure. Recent reviews emphasized the most important aspects of the electrochemical reduction of N-O containing species [123], in general, and of nitro-substituted benzamide compounds [124], in particular. CAP cyclic voltammograms usually exhibited three signals: one situated at more negative potentials, C1, which is generated by the irreversible reduction of the aryl-nitro group to the corresponding hydroxylamine and a pair of redox peaks (A2/C2) observed at less cathodic potential values, which was attributed to the reversible hydroxylamine (–NHOH)/nitroso (–NO) charge transfer equilibrium (Figure 3).

With very few exceptions, the reduction signal C1 was exploited for CAP electrochemical quantification [80]. Investigation of the pH influence on this signal emphasized that the highest peak currents were usually obtained in PBS pH 7.00 [50,56,57,58,61,65,66,67,70,80,83,84,91,92,93,110,111,112,113] or 7.40 [55,62,74,105,107], but depending on the employed working electrode somewhat lower (BRB pH 5.00 at PGE [5]) or higher (pH 8.00 at Pt-Pd NCs/rGO/GCE [68]) values were established to be optimum. This observation was correlated with CAP pKa value (5.52) [48,92] and with the fact that CAP is easily hydrolyzed in both acidic and alkaline media [92,112]. 

Despite the fact that at most reported electrodes CAP electrochemistry followed the same pathway [50,58,60,61,62,65,66,67,74,83,84,89,93,94,97,110,111,113,115], the corresponding signals were shifted within some limits of about 0.200–0.300 V as can be observed from Figure 3. On the other hand, the peak current variation with the scan rate emphasized that at some electrodes CAP reduction was diffusion controlled [5,50,57,60,61,62,74,84,89,90,91,110,111,112,113], whereas in other cases the electrode process was governed by the CAP adsorption on the sensor surface [55,65,66,67,72,83,87,94,97,105,107]. This behavior was attributed to various causes, such as a larger specific surface area [70], more active sites [115] of the modified electrode which enabled the fixation of more analyte molecules, electron-rich (N– and O– containing groups) active sites at the PDA-VGCF electrode surface that facilitate the adsorption of the nitro group [94] or to the benzene ring structure of the modifier which can adsorb more CAP due to the conjugation effect and electrostatic interactions, as it was explained for MoS_2_-IL/GO/GCE [83], MIL-101(Cr)/XC-72 [85] or P(EBT)/GCE [92]. 

However, in the literature can be found some slight deviations from the general mechanism of CAP reduction, discussed above. For example, it was observed that at CuNDs/MWCNTs/GCE the irreversible reduction of –NO_2_ to –NHOH is followed by a subsequent irreversible reduction of –NHOH to –NH_2_, which involved two electrons and two protons and generated a signal at about 0.200 V more cathodic potential [72]. The possible CAP electrochemical mechanism at CoMoO_4_/GCE included the well-known electrode processes but also a step implying the transformation of the hydroxylamine (–NHOH) into quinoneimine (=NH) by losing a water molecule [59]. 

A totally different mechanism was proposed for CAP reduction at the ZnWO_4_NWs/GCE, involving the following steps: (1) the transfer of electrons to the conduction band of the ZnWO_4_NWs; (2) these electrons reacted with water molecules and generated hydroxyl radicals and H_2_; (3) the hydroxyl radicals attacked the CAP nitro group resulting an anionic CAP derivative, which (4) dissociated into three fragments [90]. 

The best known and frequently used way to enhance the redox signals in order to improve the detection sensitivity of the electroanalytical methods is to change the properties of the electroactive electrode surface by modifying it with various materials, most of them being characterized by an electrocatalytic activity. However, signal amplification can be also obtained by the introduction of a homogeneous chemical reaction coupled with the electrode reaction. On this principle, a redox capacitor based on SPANI–CHIT composite deposited on a GCE, was developed to amplify the CAP main cathodic signal (generated by the nitro group reduction), through a redox-cycling reaction in the presence of ferrocenecarboxylic acid (Fc) acting as mediator. This can be monitored by CV measurements and briefly explained as follows: by cycling the potential, Fc is oxidized to Fc^+^ while the nitro group of CAP is, as usually, reduced to the CAP hydroxylamine derivative, which reacted with the quinone diimine (Q) moiety of the SPANI–CHIT composite, resulting in the corresponding nitroso-compound and benzenoid diamine (H_2_Q). Then, the H_2_Q was oxidized to Q by generating an anodic redox recycling using Fc^+^ as electron acceptor [95]. 

**Scheme 1:**
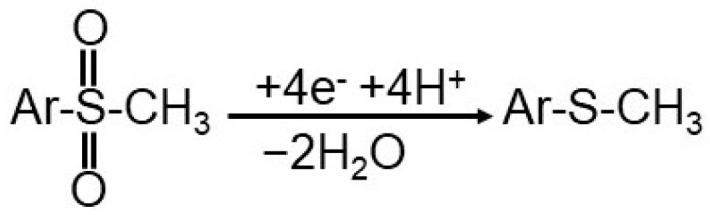
FF electroreduction reaction.

FF structure is similar to that of CAP but the electrochemically active group is different so that the methanesulfonyl group in FF is reduced, generating a cathodic signal situated at a more negative potential in comparison to –NO_2_ reduction peak of CAP. At G/CuPc/GCE FF was irreversible reduced (E_pFF_ = −0.732 V in 0.10 mol/L PBS pH 7.40) according to the following diffusion-controlled reaction [62] (Scheme 1): 

### 3.2. Electrochemical Oxidation

A recent paper described, for the first time, CAP quantification by LSV at PGE based on the anodic peak corresponding to the oxidation of the hydroxylamine group to the nitroso moiety [5]. 

Electrodes modified with nickel hydroxide are known to generate the NiOOH species responsible for the relative fast catalytic electrooxidation of hydroxyl and amino groups which are also present in CAP. Thus, CAP electrooxidation was investigated by CV in 0.10 mol/L NaOH at various GCEs modified by electrodeposition with nickel, mixed nickel/cobalt and cobalt tetraruthenated porphyrin polymeric films. The modified electrodes were called Ni-100 (pure NiTRP), Ni-75 (25% CoTRP), Ni-50 (50% CoTRP), Ni-25 (75% CoTRP) and Co-100 (100% CoTRP). In the absence of CAP all modified GCEs presented a couple of well-defined peaks attributed to the Ni^3^^+^^/2^^+^ redox processes, while after adding CAP to the analyzed solution a new oxidation signal, at more positive potentials, was observed. The currents of both, this new anodic signal and of the peak corresponding to the Ni^2+/3+^ redox couple, increased linearly with CAP concentration in the range 0.10–0.60 mmol/L, when GCEs modified with Ni-100, Ni-75 and Ni-50 films acted as working electrodes. For the electrodes with lower (Ni-25) or no Ni (Co-100) content the linear range was narrower (0.10–0.40 mmol/L) and a slight deviation from the linearity was observed due to the fact that nickel does no longer play an important role in the CAP oxidation, thus displacing its oxidation potential towards more positive values. Considering both, the best selectivity and sensitivity, Ni-50/GCE was found to be more adequate for CAP analysis [120]. 

**Scheme 2:**
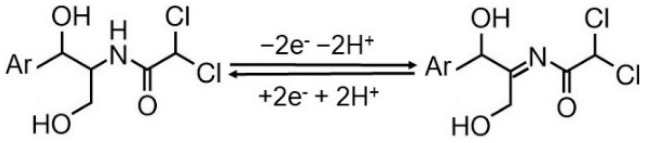
TAP electrooxidation reaction.

Thiamphenicol AdS-DPV determination at CNTs/en/AuNPs/SPE [118] was also based on its adsorption-controlled oxidation, which was considered to take place at the amide group of the aromatic (Ar) side chain according to the reaction (Scheme 2): 

### 3.3. Electrochemical Impedance Spectroscopic (EIS) Detection

CAP determination at different electrodes modified with MIP(EBT) was carried out by EIS. The detection principle was based on the fact that the incubation of the working electrode with increasing CAP concentrations resulted in a linear enhancement of the sensors’ electrical charge transfer resistance (R_ct_). R_ct_ was estimated from the diameter of the Nyquist plots obtained using a fixed concentration of [Fe(CN)_6_]^3-/4-^ as redox probe in KCl solution. The analytical performances of the electrodes modified with MIP(EBT) in the same conditions are listed in Table 2.

A very recent paper reported the use of a GCE modified with a zirconium-based metal organic framework and carbon dots composite covered with a molecularly imprinted polypyrrole film for the EIS detection of CAP in the concentration range 0.10–100.00 pmol/L with a limit of detection of 0.061 pmol/L [125]. 

Quantitative determination of FF by EIS at a MIP(5-IAA)/(Pt-Pd)NPs/P N-G/GCE was based on the same principle, the R_ct_ varying linearly with increasing FF concentrations in the range 5.00 × 10^−8^–8.00 × 10^−6^ mol/L, according to the regression equation Z’(Ω) = 1634 + 4051 C (μmol/L), R^2^ = 0.9925). The method had a limit of detection of 1.00 × 10^−9^ mol/L and was applied to FF determination in feedstuff, milk and porcine muscle [121].

### 3.4. Potentiometric Detection

Potentiometric detection is based on Nernst relation, which establishes the correlation between the measured potential and the analyte activity. Unfortunately, we found only three reports in the literature regarding the potentiometric analysis of amphenicols and they are not very new. However, they are briefly presented below. 

A very early study described an indirect potentiometric method for CAP quantification. The method principle consisted of the CAP reduction with metallic cadmium and the potentiometric titration with EDTA of the Cd^2+^ formed in the reaction using a cadmium ion-selective electrode [126]. A carbon paste electrode based on CAP-molecularly-imprinted methacrylic acid, MWCNTs, nanosilica, the ionic liquid (1-N-butyl-3-methylimidazolium tetrafluoroborate) and graphite presented a Nernstian slope of 59.1 ± 0.4 mV/decade, a linear range from 1.00 × 10^−6^ to 1.00 × 10^−2^ mol/L, a limit of detection of 1.00 × 10^−6^ mol/L CAP and it was applied to CAP determination in pharmaceutical tablets [127]. A CAP succinate ion-selective electrode with a PVC membrane containing the phosphomolybdate–CAP succinate complex as active component and di-octyl phthalate or di-butyl phthalate as plasticizer displayed responses of 58.5 mV/decade and 53.9 mV/decade, linear ranges of 1.00 × 10^−4^–1.00 × 10^−1^ mol/L and 2.00 × 10^−4^–1.00 × 10^−1^ mol/L and limits of detection of 5.50 × 10^−5^ mol/L and 8.00 × 10^−5^ mol/L CAP, respectively [128].

### 3.5. Immunosensors and Aptasensors

Electrochemical immunosensors and aptasensors are biosensors which combine the specificity and affinity of the receptor (antibody or aptamer acting as recognition element) towards the analyte with the simplicity, low cost, accurate and rapid response of the signal transducer (the bare or modified electrode) [27]. Electrochemical immunosensors have enhanced sensitivity and offer a highly improved selectivity due to the specific antibody-antigen molecular recognition reaction. Thus, CAP detection using immunosensors was based either on the increase of the current generated by CAP reduction [104] or by the decrease of the signal of a redox probe, e.g., K_3_[Fe(CN)_6_], [103] with increased concentrations of CAP that was bound to the CAP-antibody immobilized at the electrode surface. Aptasensors are electrodes modified with aptamers that are single stranded oligonucleotides with characteristic identification sites and configuration that enable a specific interaction with the analyte, thus enabling the selective accumulation of the target species. Detailed information on this topic can be found in two review papers dedicated to the CAP aptasensors based on different optical or electrochemical detection principles [33,34].

## 4. Simultaneous Electrochemical Detection of Amphenicol and Other Compounds

Some reports described the CAP voltammetric analysis in the presence of another species. The simultaneous CV responses of hydroquinone and CAP showed additional peaks of a redox couple E_pc_ = −0.180 V and E_pa_ = 0.009 V which were attributed to a possible interaction between the two analytes. The linear I_p_ = f(v^1/2^) (I_p_ represents the peak current and v the scan rate) dependencies of the hydroquinone and CAP signals obtained simultaneously when they were in a mixture, emphasized diffusion-controlled processes of both compounds at Gd_2_(MoO_4_)_3_@rG/GCE. However, the authors did not report the simultaneous quantitative analysis of the two substances [57]. 

SWV simultaneous determination of riboflavin and CAP was possible at N-PC@MoS_2_/GCE due to the fact that there was a separation of 0.272 V between the peak potentials of the two analytes. Moreover, the results obtained for both individual and simultaneous determination of the drugs were very similar [82]. DPV at NiCo_2_O_4_@C/GCE method was employed for the concomitant determination of furazolidone) and CAP in their binary mixture, based on the two reduction signals separated by a difference of 0.192 V (E_p,furazolidone_ = –0.332 V and E_p,CAP_ = 0.524 V). According to the slopes of the currents versus concentrations dependencies, obtained by keeping the concentration of one substance constant while that of the other varied within the linear range, the sensitivities for furazolidone and CAP were 1.980 and 0.575 µA/µM·cm^2^, respectively [60]. Simultaneous DPV determination of metronidazole and CAP from mixtures in KCl containing phosphate buffer solution pH 7.00 at GNFls/GCE was studied by systematically changing the concentration of the two antibiotics. The assay was possible because in these conditions metronidazole and CAP reduction signals were well-resolved, there being a peak potential (E_p,metronidazole_ = –0.160 V and E_p,CAP_ = –0.476 V) difference of 0.280 V [50]. At the Fe/NC-Nafion/GCE the LSV peaks of CAP and metronidazole shifted towards each other with increasing buffer pH, eventually merging into a single peak and therefore they were determined in acidic medium (pH 1.80). When the concentration of metronidazole was kept unchanged and CAP concentration was varied, metronidazole peak currents remained constant while those of CAP increased linearly. In turn, when the concentration of CAP was constant and that of metronidazole was increased, the peak current of metronidazole exhibited a linear relation with its concentrations but CAP reduction peak currents did not remain constant. Moreover, with increasing concentrations of both analytes, their reduction signals tended towards merging into one. However, CAP reduction peak current varied linearly, but with different slopes, in the range of 1.00 × 10^−7^–3.00 × 10^−5^ mol/L, and 3.00 × 10^−5^–1.00 × 10^−4^ mol/L [88].

## 5. Analytical Applications of the Electrochemical Sensors for Amphenicol Determination in Real Samples

**Figure 4:**
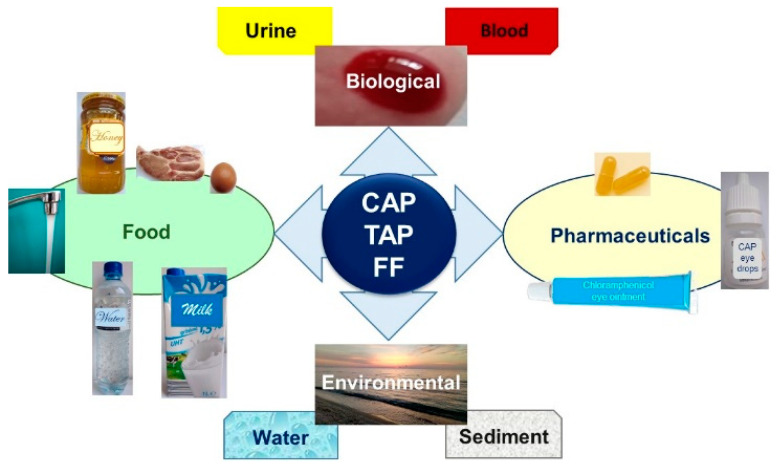
Schematic representation of samples used to test the practical ability of the sensors developed for amphenicol electrochemical analysis.

The practical applicability of the developed electrochemical sensors was tested for amphenicol determination, mainly CAP, in different matrices (Figure 4) that either contained the antibiotic, as it is the case of pharmaceuticals, or after spiking the sample with the drug of interest.

Water samples did not need any other pretreatment other than filtration and dilution with the proper supporting electrolyte. DPV analysis at g-C_3_N_4_/MnWO_4_/GCE indicated recoveries of added CAP of 92.20–98.40% in sewage and 88.40–100.20% in river water samples [89], while those obtained in tap and lake water were 93.60% and 105.70% when the Ag/CMC@TiO_2_/LIGE was used [117]. The calculated spiked recoveries of CAP from fishpond, canal and lake water samples ranged from 95.78 to 102.67% when LSV at CuNDs/MWCNTs/GCE was applied [72]. The feasibility of the PCN-222-CHIT/PEDOT/ITOE in CAP detection was demonstrated by recoveries from tap water which ranged from 95.40 to 103.10% [97]. The standard addition method was used to determine CAP in filtered and spiked tap and drinking water samples by DPV at Mn_2_O_3_@CCH/GCE, with recoveries situated between 97.00 and 99.00% [61]. 

Human blood serum and urine were usually centrifuged and the supernatant was voltammetrically investigated after dilution, most often with PBS pH 7.00. CAP was not detected in the corresponding biological samples. The recoveries of spiked CAP in serum were in the range 98.00–102.70% and 99.80–102.6% at CL-Ho^3+^/Co_3_O_4_-NFlo/GCE [91] and at N-PC@MoS_2_/GCE [82], respectively. For urine samples, the average recoveries were 96,87% at MoS_2_-IL/GO/GCE [83], 98.52% at CoMoO_4_/GCE [59], 100.23% at CL-Ho^3+^/Co_3_O_4_-NFlo/GCE [91], 101.23% at GNFls/GCE [50] and 108.13% at Fe/NC-Nafion/GCE [88]. For calf plasma, a mean CAP recovery of 102.90% was reportedly achieved using the Cl-rGO/GCE [55]. 

In order to determine the CAP content of eye drops, the samples were only diluted to bring the analyte concentration in the linear range of the applied method and the content was evaluated using the equation of the calibration curve. The results obtained by photovoltammetry at BiOI/G/GCE [63], AdS-SWV at P(EBT)/GCE [92], LSV at Fe/NC-Nafion/GCE [88], DPV at Cl-rGO/GCE [55], G/CuPc/GCE [62] and MoS_2_-IL/GO/GCE [83] were in agreement with the content claimed by the producer. AdS-SWV at P(EBT)/GCE [92] was also applied to the direct CAP quantification in eye ointment, which was diluted and filtrated prior to the analysis. Good mean CAP recoveries from eye drops were obtained using the above-mentioned sensors but also the following: Si-Fe/NOMC/GCE (99.70%) [87], MIO@NG/MSPE (104.25%) [115], MoN@S-GCN/GCE (102.00%) [84] and GO/ZnO/GCE (102.37%) [65]. 

However, as the sample matrix became more complex, pretreatment protocols were required to bring the analyte into an interference free measurable form. Some reports indicated that before the voltammetric analysis, the milk or honey samples were only diluted [59,62,65,67,110,111,112,113] whilst others also performed a centrifugation step [55,60,88,94]. The literature also contains procedures that include the addition of perchloric acid [69,81] or trichloroacetic acid [51,56,70,85,93,107] to milk samples in order to precipitate the proteins and the analyte extraction with ethyl acetate [51,56,69,70,74,81,85,93,107]. The amphenicol recoveries obtained with manifold modified sensors from different food samples are given in Table S1. Other information (such as stability, reproducibility and sample preparation protocol) regarding the sensors reported in the literature for amphenicol detection in milk samples were recently summarized by de Faria [30]. 

It is worth mentioning that a single paper reported the CAP concentrations in milk and honey samples. The results of 247.70 ± 10.80 µg/kg and 268.00 ± 12.10 µg/kg in milk and honey samples, respectively, were confirmed by HPLC analysis [93]. 

A few more examples demonstrating the analytical applications of the electrochemical sensors for the amphenicol analysis in real samples are briefly presented next. CAP measured by SWV at Fe_3_O_4_/ET-GCE [46] and DPV at Bi_2_S_3_@GCN/SPCE [113] in shrimp extracts using standard addition method gave recoveries in the range 98.70–99.10% and 99.12–99.52%, respectively. 

Meat samples were digested with acetonitrile-H_2_O solution and the diluted filtrate was analyzed by DPV at P(3-MTF)/GCE. The standard addition method was applied and the obtained FF recoveries were in the range of 105.17–111.92% for red meat and from 90.07 to 100.73% for chicken meat [119]. Recoveries between 90.00 and 104.00% for FF in feedstuff, milk and porcine muscle demonstrated the practicability of the MIP(5-IAA)/(Pt-Pd)NPs/P N-G/GCE in real samples analysis. The recovery results were confirmed by HPLC [120]. 

Poultry feed in water was treated with ethyl acetate, centrifuged and the supernatant was filtrated, dried and dissolved in the supporting electrolyte for DPV analysis at rGO@NHS@AuNFlos/SPE. The mean recovery obtained for CAP standard additions was 101.10% [107].

From Table S1 and the discussed examples it can be observed that the developed sensors tested on food samples achieved good recovery values for amphenicol. As it was expected, the declared percentage recoveries as well as the relative standard deviations were dependent on both the analyte concentration level and the sample matrix complexity, but these parameters were situated within the accepted ranges. However, it was reported that when using Fe/NC-Nafion/GCE for CAP determination from spiked fresh milk there were large differences between the added and found analyte amount due to the interference of some milk components [88].

## 6. Conclusions

Despite the fact that amphenicols have some benefits for human health, their presence above MRLs in food and environmental samples raise some concerns. Therefore, from a global security perspective there is an increasing need in the production of devices capable of accurately detecting low levels of amphenicols in complex matrices. On the other hand, the pharma research field is continuously expanding and this also implies an increased demand for reliable, rapid and cost-effective methods for the quality control of pharmaceutical dosage forms. Compared with other amphenicol detection methods the electrochemical ones are simpler, faster and can use miniaturized portable devices for in situ monitoring. Because the electrode is the core part of an electrochemical instrument, this review was centered on the electrochemical sensors developed over time for amphenicol analysis, with the most attention being paid to those reported in the last five years. 

In the field of electrochemical sensors, the research is focused mainly on the development of modified electrodes, with improved sensitivity and selectivity. Therefore, we have summarized and briefly presented comparatively the modification methods and materials of amphenicol sensitive electrodes. Regarding the amphenicol electrochemical detection principle in most cases it was a voltammetric one, based on CAP and FF reduction, but there were also few studies exploiting FF or CAP oxidation signals. However, EIS or potentiometric methods were also used in some determinations. 

In the case of complex matrices, like foods, the sample preparation step within an analytical method is as important as the measuring method and therefore the pretreatment procedures were also discussed. On the other hand, the amphenicol concentrations in food and environmental samples are very low and therefore sensitive detection devices and methods must be designed. Most of the modified electrodes reported in the last years for amphenicol quantification had limits of detection in the nanomolar range and good recoveries in real samples, but further improvement in the sensor’s sensitivity and selectivity is still of interest. As this review is a comprehensive up to date presentation of electrochemical sensors for amphenicol detection, it is expected that the representative examples of the opportunities and applications of these devices provided here may inspire interested researchers to further innovation in the field.

## Figures and Tables

**Table 1:** The performance characteristics of electrochemical sensors reported in the literature for amphenicol determination.

Electrode	Technique	Linear Range *	Limit of Detection *	Sample	Ref.
Chloramphenicol (CAP)					
DME	DPP	<3.20 × 10^−5^	200 ppb	milk	[35]
Pt	DPV	0.80–30.00 **	10.00 **	Pharmaceutical formulations, spiked milk samples	[36]
Au	SWV	2.50 × 10^−6^–7.40 × 10^−6^	1.00 × 10^−6^	–	[37]
BDDE	FIA-AD	1.00 × 10^−7^–5.00 × 10^−5^	3.00 × 10^−8^	Eye drops	[38]
CPE	DPV	1.00 × 10^−6^–1.00 × 10^−5^	5.00 × 10^−7^	–	[39]
CFME	SWV	1.00 × 10^−7^–1.00 × 10^−5^	4.70 × 10^−8^	Milk	[40]
BM–PCE	DPV	1.00 × 10^−6^–4.00 × 10^−5^ 5.00 × 10^−5^–5.00 × 10^−4^	1.00 × 10^−8^	–	[41]
ET–GCE	SWV	1.00 × 10^−7^–7.00 × 10^−5^	6.00 × 10^−9^	Eye drops	[42]
ET–GCE	SWV	1.60 × 10^−6^–2.00 × 10^−4^	2.30 × 10^−6^	Eye drops, oral suspension	[43]
GCE in presence of CTAB	LSV	2.60 × 10^−3^ – 8.00 ***	8.30 × 10^−4^ ***	Milk	[44]
Ni/GCE	LSV	1.00 × 10^−5^–1.00 × 10^−3^	5.00 × 10^−6^	Eye drops	[45]
Fe_3_O_4_/ET–GCE	SWV	9.00 × 10^−8^–4.70 × 10^−5^	9.00 × 10^−8^	Shrimp extract	[46]
Fe_3_O_4_mNPs/CFME	DPV	4.00 × 10^−11^–1.00 × 10^−6^	1.70 × 10^−11^	Spiked sediment	[47]
AuNPs/BDDE	SWV	5.00 × 10^−6^–3.50 × 10^−5^	5.00 × 10^−6^	–	[48]
c–SWCNH/GCE	–	1.00 × 10^−7^–1.00 × 10^−4^	1.00 × 10^−7^	–	[49]
GNFls/GCE	DPV Amp (–0.476 V)	1.00 × 10^−8^–2.70 × 10^−7^ 5.00 × 10^−10^–5.50 × 10^−9^	4.40 × 10^−9^ 3.80 × 10^−10^	Urine	[50]
EPC/GCE	SWV	1.00 × 10^−8^–1.00 × 10^−6^ 1.00 × 10^−6^–4.00 × 10^−6^	2.90 × 10^−9^	Honey	[51]
ENC–800/GCE	SWV	5.00 × 10^−8^–1.00 × 10^−4^ 1.00 × 10^−4^–1.00 × 10^−4^	4.00 × 10^−8^	Honey, milk, domestic sewage	[52]
rGO/GCE	AdS-DPV	–	2.20 × 10^−7^	Milk	[53]
3D_rGO/GCE	DPV	1.00 × 10^−6^–1.13 × 10^−4^	1.50 × 10^−7^	Eye drops, milk	[54]
Cl–rGO/GCE	DPV	2.00 × 10^−6^–3.50 × 10^−5^	1.00 × 10^−6^	Calf plasma, tap water, milk, eye drops	[55]
Z–800@rGO/GCE	DPV	1.00 × 10^−6^–1.80 × 10^−4^	2.50 × 10^−7^	Milk, honey	[56]
Gd_2_(MoO_4_)_3_@rG/GCE	Amp (−0.680 V)	2.00 × 10^−8^–9.00 × 10^−8^	6.30 × 10^−9^	–	[57]
Co_3_O_4_@rGO/GCE	CV DPV Amp	1.00 × 10^−6^–2.00 × 10^−3^ 2.00 × 10^−6^–2.00 × 10^−3^ 1.00 × 10^−7^–1.50 × 10^−3^	5.50 × 10^−7^ 1.16 × 10^−6^ 1.00 × 10^−7^	Milk, honey	[58]
CoMoO_4_/GCE	DPV	6.00 × 10^−8^–1.19 × 10^−3^	1.40 × 10^−8^	Milk, urine	[59]
NiCo_2_O_4_@C/GCE	DPV	5.00 × 10^−7^–3.20 × 10^−4^	3.50 × 10^−8^	Milk, honey	[60]
Mn_2_O_3_@CCH/GCE	DPV	5.00 × 10^−9^–7.94 × 10^−6^ 1.39 × 10^−5^–9.19 × 10^−5^	3.00 × 10^−8^	Tap and drinking water	[61]
G/CuPc/GCE	DPV	1.00 × 10^−7^–2.00 × 10^−5^	2.70 × 10^−8^	Eye drops, milk	[62]
BiOI/G/GCE	PhV	5.00 × 10^−7^–5.00 × 10^−5^	1.40 × 10^−7^	Eye drops, environmental water	[63]
TiN–rGO/GCE	DPV	5.00 × 10^−8^–1.00 × 10^−4^	2.00 × 10^−8^	Eye drops	[64]
GO/ZnO/GCE	DPV	2.00 × 10^−7^–7.20 × 10^−6^	1.00 × 10^−8^	Eye drops, milk	[65]
GO/PdNPs/GCE	Amp (−0.540 V)	7.00 × 10^−9^–1.03 × 10^−4^	1.00 × 10^−9^	Milk, urine	[66]
rGO/PdNPs/GCE	DPV	5.00 × 10^−8^–1.00 × 10^−6^	5.00 × 10^−8^	Tap water, honey	[67]
Pt–Pd NCs/rGO/GCE	LSV	2.00 × 10^−7^–3.00 × 10^−5^	1.00 × 10^−7^	Milk	[68]
AuNPs/GO/GCE	Amp (−0.450 V)	1.50 × 10^−6^–2.95 × 10^−6^	2.50 × 10^−7^	Eye drops, milk, honey	[69]
AuNPs/C_3_N_4_/G/GCE	SWV	7.00 × 10^−7^–1.20 × 10^−4^	2.70 × 10^−8^	Milk	[70]
AuNPs/N–G/GCE	AdS-LSV	2.00 × 10^−6^–8.00 × 10^−5^	5.90 × 10^−7^	Eye drops	[6]
OMIMPF_6_/AuNPs/SWCNTs/GCE	AdS-LSV	1.00 × 10^−8^–6.00 × 10^−6^	5.00 × 10^−9^	Milk	[71]
CuNDs/MWCNTs/GCE	LSV	1.50 × 10^−7^–1.20 × 10^−5^	9.84 × 10^−9^	Environmental water	[72]
AgNPs/S-f–G/GCE	AdS-DPV	2.00 × 10^−8^–2.00 × 10^−5^	1.00 × 10^−8^	Shrimp	[73]
MIP(MAA)/3D_CNTs@CuNPs/GCE	CV	1.00 × 10^−5^–5.00 × 10^−4^	1.00 × 10^−5^	Milk	[74]
MWCNTs/CTAB/PDPA/GCE	AdS-DPV	1.00 × 10^−8^–1.00 × 10^−5^	2.00 × 10^−9^	Milk, honey	[75]
MWCNTs@MIP/P–rGO/CKM–3/GCE	DPV	5.00 × 10^−9^–4.00 × 10^−6^	1.00 × 10^−10^	Milk, honey	[76]
OMC/Nafion/GCE	AdS-LSV	5.00 × 10^−7^–6.00 × 10^−5^	8.50 × 10^−9^	Honey	[77]
MoS_2_/PANI/CPE	DPV	1.00 × 10^−7^–1.00 × 10^−4^	6.90 × 10^−8^	honey	[78,79]
MoS_2_–rGO/GCE MoS_2_–MWCNTs/GCE MoS_2_–CB/GCE	DPV	5.00 × 10^−6^–3.50 × 10^−5^ 1.00 × 10^−6^–3.50 × 10^−5^ 5.00 × 10^−6^–5.50 × 10^−5^	1.00 × 10^−6^ 4.00 × 10^−7^ 1.90 × 10^−6^	-	[80]
MoS_2_/f–MWCNTs/GCE	Amp (−0.520 V)	8.00 × 10^−8^–1.39 × 10^−3^	1.50 × 10^−8^	Milk, powdered milk, honey	[81]
N–PC@MoS_2_/GCE	SWV	1.00 × 10^−5^–5.00 × 10^−4^	2.03 × 10^−8^	Human serum	[82]
MoS_2_–IL/GO/GCE	DPV	1.00 × 10^−7^–4.00 × 10^−4^	4.70 × 10^−8^	Eye drops, milk, urine	[83]
MoN@S–GCN/GCE	DPV	5.00 × 10^−7^–2.45 × 10^−3^	6.90 × 10^−9^	Eye drops, milk	[84]
MIL–101(Cr)/XC–72/GCE	DPV	1.00 × 10^−8^–2.00 × 10^−5^	1.50 × 10^−9^	Eye drops, honey, milk	[85]
Fe_3_O_4_–CMC@AuNPs/GCE	SWV	2.50 × 10^−6^–2.50 × 10^−5^	6.60 × 10^−8^	Human urine	[86]
Si–Fe/NOMC/GCE	DPV	1.00 × 10^−6^–5.00 × 10^−4^	3.00 × 10^−8^	Eye drops	[87]
Fe/NC–Nafion/GCE	LSV	1.00 × 10^−7^–1.00 × 10^−4^	3.10 × 10^−8^	Milk, urine	[88]
g–C_3_N_4_/MnWO_4_/GCE	DPV	4.00 × 10^−9^–7.10 × 10^−8^	1.03 × 10^−9^	Milk, human blood serum, sewage, river samples	[89]
ZnWO_4_NWs/GCE	CV	5.00 × 10^−5^–5.00 × 10^−4^	3.20 × 10^−7^	–	[90]
CL–Ho^3+^/Co_3_O_4_–NFlos/GCE	DPV	1.00 × 10^−8^–8.00 × 10^−6^	7.10 × 10^−9^	Human blood serum, urine, eye drops	[91]
P(EBT)/GCE	AdS-SWV	1.00 × 10^−8^–4.00 × 10^−6^	3.00 × 10^−9^	Eye drops, ointments	[92]
β–CD/CMK-3@PDA /GCE	SWV	5.00 × 10^−7^–5.00 × 10^−4^	2.00 × 10^−7^	Milk, milk powder, bee pollen, honey	[93]
PDA–VGCF/GCE	DPV	1.00 × 10^−8^–1.42 × 10^−4^	3.00 × 10^−9^	Milk, honey, apple juice	[94]
SPANI–CHIT/GCE	CV	5.00 × 10^−7^–5.00 × 10^−5^	1.00 × 10^−7^	Eye drops	[95]
MoS_2_/SDPANI/CPE	DPV	1.00 × 10^−7^–1.00 × 10^−3^	6.50 × 10^−8^	Eye drops	[96]
PCN–222–CHIT/ PEDOT/ITOE	AdS-DPV	1.00 × 10^−8^–8.00 × 10^−7^	1.80 × 10^−9^	Tap water	[97]
CSM@VSM/ITOE	DPV	0.10–3.60 ** 3.60–15.00 **	40 ****	Milk, honey	[98]
Apt/[NH_2_–Si]–f–GO/AgNPs/GCE	DPV	1.00 × 10^−11^–2.00 × 10^−7^	3.30 × 10^−12^	Milk, honey	[7]
Apt–MIP(Res)/AgNPs/3–ampy–rGO/GCE	EIS	1.00 × 10^−12^–1.00 × 10^−9^	3.00 × 10^−13^	Milk	[99]
SSB/CAP/MCH/Apt/PEI–rGO/AuNCs/AuE	DPV	5.00 × 10^−12^–1.00 × 10^−6^	2.08 × 10^−12^	Chicken meat	[100]
Apt/PCN–222/GO/AuE	EIS	1.00 × 10^−11^–5.00 × 10^−8^	7.04 × 10^−12^		[101]
tDNA-Apt/SPAuE	DPV	3.00 × 10^−10^–2.00 × 10^−9^	1.83 × 10^−10^	Milk	[102]
Anti–CAP/HGNS/CHIT/GCE	DPV	0.10–1000.00 ****	0.06 ****	Fish, beef and pork meat	[103]
Anti–CAP/PVA–co–PE NFM/SPCE	Amp (−0.660 V)	0.01–10.00 ****	0.0047 ****	Milk	[104]
SPCE SPPtE AuNPs/SPCE AuNPs/SPPtE	DPV	1.00 × 10^−6^–5.00 × 10^−5^ 2.50 × 10^−7^–5.00 × 10^−5^	1.00 × 10^−6^ 1.36 × 10^−6^ 1.00 × 10^−7^ 6.60 × 10^−7^	–	[105]
rGO/Cu_2_S NS/SPCE	Amp (−0.720 V)	6.00 × 10^−8^–1.95 × 10^−3^	1.27 × 10^−8^	Milk (fresh, powder), ice cream	[106]
rGO@NHS@AuNFlos/SPE	DPV	5.00 × 10^−8^–1.00 × 10^−4^	1.00 × 10^−9^	Blood serum, milk, powdered milk, honey, eggs, poultry feed	[107]
MIP(EBT)/SPCE	SWV SWV	1.00 × 10^−9^–1.00 × 10^−4^	6.53 × 10^−10^ 6.53 × 10^−10^	Home fish aquarium water	[108]
MIP(MAA)/SPE	CV	1.00 × 10^−8^–1.20 × 10^−5^	2.00 × 10^−9^	Milk	[109]
Mn_2_O_3_TNSs/SPCE	DPV	1.50 × 10^−8^–5.66 × 10^−4^	4.26 × 10^−9^	Milk	[110]
Eu_2_O_3_NPs@rGO/SPCE Eu_2_O_3_NPs@rGO/RE	CV Amp (0.420 V)	5.00 × 10^−5^–2.50 × 10^−4^ 2.00 × 10^−8^–8.00 × 10^−4^	1.32 × 10^−9^	Fresh milk, honey	[111]
Sr–ZnO@rGO/SPCE	LSV	1.90 × 10^−7^–4.11 × 10^−4^	1.31 × 10^−7^	Milk, powdered milk	[112]
Bi_2_S_3_@GCN/SPCE	DPV	2.00 × 10^−8^–3.74 × 10^−4^	1.20 × 10^−9^	Fresh milk, shrimps, honey	[113]
Fe_3_O_4_@G/MSPE	CV	5.00 × 10^−5^–5.00 × 10^−4^	1.70 × 10^−5^	–	[114]
MIO@NG/MSPE	SWV	1.00 × 10^−8^–2.00 × 10^−6^ 5.00 × 10^−6^–2.00 × 10^−4^	1.00 × 10^−8^	Milk (powder and bottled), eye drops	[115]
PGE	LSV SWV	2.50 × 10^−6^–1.00 × 10^−3^ 2.50 × 10^−6^–7.50 × 10^−4^	6.09 × 10^−7^ 1.39 × 10^−6^	Pharmaceutical capsules	[5]
FGE	DPV	1.00 × 10^−5^–2.00 × 10^−4^	2.70 × 10^−6^	Pork meat, milk	[116]
Ag/CMC@TiO_2_/LIGE	DPV	1.00 × 10^−8^–1.00 × 10^−4^	7.00 × 10^−9^	Tap and lake water	[117]
Thiamphenicol (TAP)					
CNTs/en/AuNPs/SPCE	AdS-DPV	1.00 × 10^−7^–1.00 × 10^−5^ 1.00 × 10^−5^–3.00 × 10^−5^	3.00 × 10^−9^	Milk	[118]
Florfenicol (FF)					
P(3–MTF)/GCE	DPV	1.00 × 10^−4^–1.00 × 10^−3^	3.99 × 10^−5^	Red and chicken meat	[119]
G/CuPc/GCE	DPV	1.00 × 10^−6^–3.00 × 10^−5^	7.50 × 10^−7^	Milk	[62]

* mol/L if not stated otherwise; ** μg/mL; *** mg/mL; **** ng/mL.

**Table 2:** Comparative performance characteristics of MIP(EBT) modified carbon-based sensors for EIS detection of CAP.

Sensor Support	Linear Range (mol/L)	Sensitivity (Ω/Decade)	RSD (%) ^1^	Limit of Detection (mol/L)	Sample	Ref.
SPCE	1.00 × 10^−9^–1.00 × 10^−4^	100.80	3.41	2.60 × 10^−10^	Home fish aquarium water	[108]
SPCE	1.00 × 10^−9^–1.00 × 10^−4^	103.71	2.70			
SPGE	1.00 × 10^−9^–1.00 × 10^−3^	77.00	1.42			[122]
LIGE	1.00 × 10^−9^–1.00 × 10^−2^	162.50	3.18	6.20 × 10^−10^		

^1^ relative standard deviation.

## Data Availability

Not applicable.

## Supplementary Materials

The following supporting information can be downloaded at: https://www.mdpi.com/article/10.3390/mi13050677/s1, Table S1. The recoveries obtained for amphenicol from common food samples.

## Abbreviations

3D_rGO	three–dimensional reduced graphene oxide;
AdS–DPV	adsorptive stripping differential pulse voltammetry;
AdSV	adsorptive stripping voltammetry;
AdS–LSV	adsorptive stripping linear sweep voltammetry;
AdS–SWV	adsorptive stripping square wave voltammetry;
Ag/CMC@TiO_2_/LIGE	TiO_2_ modified with sodium carboxymethyl cellulose and silver nanoparticles deposited onto laser induced graphene electrode;
AgNPs/S-f–G	silver nanoparticles/sulfonate functionalized graphene;
Amp	amperometry;
Anti–CAP/HGNS/CHIT	CAP antibody/hollow gold nanospheres/chitosan;
Anti–CAP/PVA–co–PE NFM	CAP antibody covalently immobilized on poly(vinyl alcohol-co-ethylene) nanofibrous membrane;
Apt–MIP(Res)/AgNPs/3–ampy–rGO	aminoaptamer-molecularly imprinted polyresorcinol/silver nanoparticles/3–aminoethyl functionalized reduced graphene oxide;
Apt/[NH_2_–Si]–f–GO/AgNPs	aptamer/graphene oxide functionalized with (3-aminopropyl) triethoxysilane/silver nanoparticles;
Apt/PCN–222/GO/AuE	aptamer–zirconium–porphyrin metalorganic framework/graphene oxide modified gold electrode;
AuNPs	gold nanoparticles;
AuNPs/C_3_N_4_/G	gold nanoparticles/carbon nitride/graphene;
AuNPs/GO	gold nanoparticles/graphene oxide;
AuNPs/N–G	gold nanoparticles decorated nitrogen–doped graphene;
BDD(E)	boron doped diamond (electrode);
BiOI/G	bismuth oxyiodide/graphene;
Bi_2_S_3_@GCN	bismuth trisulfide–graphitic carbon nitride hybrid-based core–shell nanomaterials;
BM–PCE	biomass derived porous carbon electrode;
β–CD/CMK-3@PDA	β–cyclodextrin/ordered mesoporous carbon@polydopamine;
CF(ME)	carbon fiber (microelectrode);
Cl–rGO	chlorine doped reduced graphene oxide;
CL–Ho^3+^/Co_3_O_4_-NFlos	carnation like holmium doped Co_3_O_4_ nanoflowers;
c-SWCNH	carboxylic group-functionalized single–walled carbon nanohorns;
CNTs/en/AuNPs	carbon nanotubes/ethylenediamine/gold nanoparticles;
Co_3_O_4_@rGO	cobalt oxide nanocrystals on reduced graphene oxide;
CP(E)	carbon paste (electrode);
CSM@VSM	cylindrical surfactant micelles and vertical silica mesochannels;
CTAB	cetyltrimethylammonium bromide;
CuNDs/MWCNTs	copper nanodendrites/multi–walled carbon nanotubes;
CV	cyclic voltammetry;
DME	dropping mercury electrode;
DMF	dimethylformamide;
DPP	differential pulse polarography;
DPV	differential pulse voltammetry;
EIS	electrochemical impedance spectroscopy;
ENC–800	exfoliated metalorganic framework–derived N–doped honeycomb cavernous carbon;
EPC	exfoliated porous carbon;
Eu_2_O_3_NPs@rGO	Eu_2_O_3_ nanoparticles decorated reduced graphene oxide;
ET	electrochemically pretreated;
Fe/NC	nitrogen doped carbon nanoparticles decorated with iron;
Fe_3_O_4_–CMC@AuNPs	magnetite nanostructures stabilized with carboxymethyl cellulose and decorated with gold nanoparticles;
Fe_3_O_4_@G/MSPE	graphene-iron oxide nanoparticles modified magnetic screen-printed electrode;
Fe_3_O_4_mNPs	Fe_3_O_4_ magnetic nanoparticles;
FGE	flexible graphene electrode;
FIA-AD	flow injection analysis with amperometric detection;
g–C_3_N_4_/MnWO_4_	graphitic carbon nitride/manganese tungstate;
G	graphene;
Gd_2_(MoO_4_)_3_@rG	gadolinium molybdate nanosheets–reduced graphene nanocomposite;
G/CuPc	graphene–copper phthalocyanine;
GC(E)	glassy carbon (electrode);
GNFls	graphene nanoflakes;
GO	graphene oxide;
GO/ZnO	graphene oxide/three-dimensional hierarchical zinc oxide nanocomposite;
GO/PdNPs	palladium nanoparticles decorated with graphene oxide;
ITO(E)	indium tin oxide (electrode);
LIG(E)	laser induced graphene (electrode);
LSV	linear sweep voltammetry;
MIL–101(Cr)/XC-72	Material Institute Lavoisier–101(Cr)—a metalorganic framework constructed by chromium ion and terephthalate ligands/a kind of carbon black hybrid;
MIO@NG	magnetic iron oxide embed nitrogen–doped graphene nanohybrid;
MIP	molecularly imprinted polymer;
MIP(EBT)	molecularly imprinted poly(Eriochrome black T);
MIP(5-IAA)/(Pt-Pd)NPs/P N-G	molecularly imprinted poly(indole-5-carboxylic acid)/dendritic platinum-palladium bimetallic nanoparticles/porous N-doped graphene;
MIP(MAA)	molecularly imprinted poly(methacrylic acid);
MIP(MAA)/3D_CNTs@CuNPs	molecularly imprinted poly(methacrylic acid)/three–dimensional carbon nanotubes–copper nanoparticles composite;
Mn_2_O_3_@CCH	manganese oxide supported on carbon modified halloysite nanotube composite;
Mn_2_O_3_TNSs	manganese oxide tiny nanostructures;
MoN@S–GCN	molybdenum nitride nanorods sulfur–doped graphitic carbon nitride nanocomposite;
MoS_2_–CB	molybdenum disulfide–carbon black;
MoS_2_/f–MWCNTs	molybdenum disulfide nanosheets coated on functionalized multiwalled carbon nanotubes;
MoS_2_–IL/GO	MoS_2_–ionic liquids/graphene oxide;
MoS_2_/PANI	molybdenum disulfide/polyaniline nanocomposite;
MoS_2_/SDPANI	MoS_2_ intercalated by self-doped polyaniline;
MRLs	maximum residue limits;
MRPL	minimum required performance limit;
MS	mass spectrometry;
MWCNT	multiwalled carbon nanotube;
MWCNTs/CTAB/PDPA	multiwalled carbon nanotubes/cetyltrimethylammonium bromide/ poly(diphenylamine);
MWCNTs@MIP/P–rGO/CKM–3	multiwalled carbon nanotubes –molecularly imprinted polymer/porous reduced graphene oxide/mesoporous carbon;
NiCo_2_O_4_@C	hollow NiCo_2_O_4_ and carbon composite;
NC	nanocube;
ND	nanodendrite;
NF	nanofiber;
NFl	nanoflake;
NFlo	nanoflower;
NPs	nanoparticle;
N–PC@MoS_2_	MoS_2_ nanosheets on nitrogen doped seaweed–derived porous carbon;
NW	nanowire;
OMC/Nafion	ordered mesoporous carbon/Nafion composite film;
OMIMPF_6_/AuNPs/SWCNTs	1–octyl–3–methylimidazolium hexafluorophosphate film/gold nanoparticles/single–walled carbon nanotubes composite;
P(3-MTF)	poly(3-methyltiophene);
PC(E)	porous carbon (electrode);
PCN–222–CHIT/PEDOT	zirconium–based porphyrinic metalorganic frameworks—chitosan/poly 3,4–ethylenedioxythiophene;
PDA-VGCF	polydopamine functionalized vapor-grown carbon fiber;
P(EBT)	poly (Eriochrome black T);
PEDOT	poly(3,4–ethylenedioxythiophene);
PG(E)	pencil graphite (electrode);
PhV	photovoltammetry;
RE	rotating electrode;
Pt–Pd NCs/rGO	bimetallic alloyed Pt–Pd nanocubes supported on reduced graphene oxide nanosheets;
rGO	reduced graphene oxide;
rGO/Cu_2_S NS	copper sulfite nanosphere decorated reduced graphene oxide;
rGO@NHS@AuNFlos	reduced graphene oxide crosslinked with N-hydroxysuccinimide and functionalized with gold nanoflowers;
rGO/PdNPs	palladium nanoparticles decorated reduced graphene oxide;
Si–Fe/NOMC	iron–nitrogen co–doped ordered mesoporous carbon–silicon nanocomposite;
SPANI–CHIT	sulfonated polyaniline–chitosan composite;
SPE	screen printed electrode;
SPCE	screen printed carbon electrode;
SPGE	screen printed graphene electrode;
SPPtE	screen printed platinum electrode;
Sr–ZnO@rGO	strontium doped zinc oxide–reduced graphene oxide nanocomposite;
SSB/CAP/MCH/Apt/PEI–rGO/AuNCs/AuE	6–mercapto–1–hexanol treated SH–aptamer polyethyleneimine–functionalized reduced graphene oxide/gold nanocubes modified gold electrode exposed to CAP and incubated with single-stranded DNA binding protein;
SWCNT	single walled nanotube carbon;
SWV	square wave voltammetry;
tDNA-Apt/SPAuE	thiolated DNA aptamer/screen printed gold electrode;
TiN–rGO	titanium nitride–reduced graphene oxide;
TRP	tetraruthenated porphyrin;
UV-Vis	ultraviolet-visible;
Z–800@rGO	porous carbon material Z-800 obtained from the zeolitic imidazolate framework ZIF–8–reduced graphene oxide nanocomposite;
ZnWO_4_NWs	zinc tungstate nanowires.
