# Supplementary material for: Past and Present of Electrochemical Sensors and Methods for Amphenicol Antibiotic Analysis
Source: Micromachines (Basel). 2022 Apr 27;13(5):677. doi: 10.3390/mi13050677 (PMC9143398; doi:10.3390/mi13050677)
Supplement: Supplementary file 1 [file micromachines-13-00677-s001.zip › micromachines-1665282-supplementary.pdf]

Supplementary information

Past and Present of Electrochemical Sensors and Methods for Amphenicol Antibiotic Analysis

Iulia Gabriela David *, Mihaela Buleandra, Dana Elena Popa, Mihaela Carmen Cheregi * and Emilia Elena Iorgulescu

Department of Analytical Chemistry, Faculty of Chemistry, University of Bucharest, Panduri Av. 90-92,
District 5, 050663 Bucharest, Romania; mihaela.buleandra@g.unibuc.ro (M.B.); elena.popa@chimie.unibuc.ro (D.E.P.); emilia-elena.iorgulescu@chimie.unibuc.ro (E.E.I.)
* Correspondence: gabrielaiulia.david@g.unibuc.ro (I.G.D.); mihaela.cheregi@g.unibuc.ro (M.C.C.)

Table S1. The recoveries obtained for amphenicol from common food samples.

**Electrode**	**Mean recovery (%)**							**Ref.**
	Milk		Honey	Eggs	Tap water	Apple juice	Ice cream	
	fresh	powder						
		**CAP**						
MoS_2/f–MWCNTs/GCE	97.35	96.05	96.60					[81]
AuNPs/C_3N_4/G/GCE	96.60							[70]
β -CD/CMK-3@PDA/GCE		96.20	Bee pollen 85.33					[93]
rGO@NHS@AuNFlos/SPE	95.60		101.30	103.30				[107]
Z-800/rGO/GCE	103.70		100.65					[56]
EPC/GCE			102.00					[51]
MIL-101(Cr)/XC-72/GCE	98.50		98.00					[85]
MIP(MAA)/3D_CNTs@CuNPs/GCE	94.16							[74]
Co_3O_4@rGO/GCE	97.90	97.90	97.77					[58]
rGO/Cu_2S NS/SPCE	98.77	98.80					98.23	[106]
G/CuPc/GCE	98.03							[62]
$CoMoO_4$/GCE	98.32							[59]
Mn_2O_3 TNSs/ SPCE	96.75							[110]
Bi_2S_3@GCN/SPCE	99.39		99.33					[113]
Apt-MIP(Res)/AgNPs/3-ampy-rGO/GCE	100.50							[99]
rGO/PdNPs/GCE			93.00		106.60			[67]
GO/ZnO/GCE	94.41		99.33					[65]
Eu_2O_3NPs@rGO/SPCE	98.24		98.48					[111]
Sr-ZnO@rGO/SPCE	99.03	98.12						[112]
MIO@NG/MSPE	100.15	100.8						[115]
MoN@S-GCN/GCE		98.75						[81]
Cl-rGO/GCE	102.95				102.15			[55]
PDA-VGCF/GCE	99.36		97.84			98.28		[94]
NiCo2O4@C/GCE	100.97		99.52					[60]

ENC-800/GCE	98.33	100.33	[52]
**FF**			
G/CuPc/GCE	98.60		[61]
**TAP**			
CNTs/en/AuNPs/SPCE	99.95		[118]
